# Newborn screening for citrin deficiency and carnitine uptake defect using second-tier molecular tests

**DOI:** 10.1186/1471-2350-14-24

**Published:** 2013-02-10

**Authors:** Li-Yun Wang, Nien-I Chen, Pin-Wen Chen, Shu-Chuan Chiang, Wuh-Liang Hwu, Ni-Chung Lee, Yin-Hsiu Chien

**Affiliations:** 1 Graduate Institute of Molecular Medicine, National Taiwan University College of Medicine, Taipei, Taiwan; 2 Department of Medical Genetics and Pediatrics, National Taiwan University Hospital and National Taiwan University College of Medicine, 7 Chung-Shan South Road, Taipei 10016, Taiwan; 3 Current address: Taipei Institute of Pathology, Taipei, Taiwan

**Keywords:** Newborn screening, Founder mutation, Second-tier molecular test, Citrin deficiency, Carnitine uptake defect

## Abstract

**Background:**

Tandem mass spectrometry (MS/MS) analysis is a powerful tool for newborn screening, and many rare inborn errors of metabolism are currently screened using MS/MS. However, the sensitivity of MS/MS screening for several inborn errors, including citrin deficiency (screened by citrulline level) and carnitine uptake defect (CUD, screened by free carnitine level), is not satisfactory. This study was conducted to determine whether a second-tier molecular test could improve the sensitivity of citrin deficiency and CUD detection without increasing the false-positive rate.

**Methods:**

Three mutations in the *SLC25A13* gene (for citrin deficiency) and one mutation in the *SLC22A5* gene (for CUD) were analyzed in newborns who demonstrated an inconclusive primary screening result (with levels between the screening and diagnostic cutoffs).

**Results:**

The results revealed that 314 of 46 699 newborns received a second-tier test for citrin deficiency, and two patients were identified; 206 of 30 237 newborns received a second-tier testing for CUD, and one patient was identified. No patients were identified using the diagnostic cutoffs. Although the incidences for citrin deficiency (1:23 350) and CUD (1:30 000) detected by screening are still lower than the incidences calculated from the mutation carrier rates, the second-tier molecular test increases the sensitivity of newborn screening for citrin deficiency and CUD without increasing the false-positive rate.

**Conclusions:**

Utilizing a molecular second-tier test for citrin deficiency and carnitine transporter deficiency is feasible.

## Background

Tandem mass spectrometry (MS/MS) analysis is a powerful tool for newborn screening [1]. Many rare inborn errors of metabolism that were not covered by newborn screening are now screened by MS/MS. However, the sensitivity of MS/MS screening for the 20–30 diseases included in its screening panel varies among the individual diseases [2]. False negatives can also occur [3,4], due to the nature of the specific diseases. Therefore, additional or second-tier testing may be required to improve screening for these diseases [5].

Citrin deficiency refers to two disease entities: adult-onset type II citrullinemia (CTLN2, OMIM#603471) and neonatal intrahepatic cholestasis caused by citrin deficiency (NICCD, OMIM#605814). Citrin is a mitochondrial membrane aspartate-glutamate carrier that functions as part of the malate-aspartate (MA) shuttle, transferring cytosolic NADH into the mitochondria of the liver [6]. Patients with NICCD often present with jaundice, hypoproteinemia, transient multiple aminoacidemia (citrulline, methionine, tyrosine, threonine), fatty liver, galactosemia, hypoglycemia, disturbed coagulation, and high α-fetoprotein [6,7]. Citrin deficiency can be detected by newborn screening as an elevation of phenylalanine, methionine, or galactose levels, but the detection rate is only 50% [8]. It has been shown that 1 in 20 newborns affected with NICCD have normal dried blood spot (DBS) citrulline levels initially, but these levels increase at later time points [9].

Carnitine uptake defect (MIM #212140; CUD), also known as primary carnitine deficiency, is an autosomal recessive disorder of fatty acid oxidation caused by defects in OCTN2, a high-affinity carnitine transporter expressed at the plasma membrane [10,11]. Carnitine is responsible for transporting fatty acids into the mitochondria, and defective carnitine uptake results in an intracellular carnitine deficiency, causing defects in the β-oxidation of fatty acids [12]. Patients with CUD can suffer from cardiomyopathy, muscle weakness, recurrent hypoketotic hypoglycemic coma, Reye-like syndrome, and premature death [13-15]. Because most symptoms are reversible, early treatment of the disease can result in a good patient outcome [12,16]. CUD can be detected in newborns by measuring free carnitine levels in DBS [17]. However, because carnitine is transported through the placenta [18], carnitine can be supplemented by the mother, resulting in only half of fetuses with CUD being detected by newborn screening [19]. Additionally, a normal fetus can have low free carnitine levels due to carnitine deficiency in the mother [17].

The molecular defects associated with the aforementioned diseases have been elucidated. Citrin deficiency is caused by mutations in the *SLC25A13* gene [20]. A study of 4 169 normal Chinese individuals revealed four *SLC25A13* mutations: c.851_854del (851del4, p.M285PfsX2) (70%), c.1638_1660dup23 (1638ins23, p.A554GfsX17) (5%), IVS6+5 G>A (23%), and c.550C>T (p.R184X) (2%), and the total carrier rate was 1 in 65 [21]. The *SLC22A5* gene encodes the carnitine transporter OCTN2 [10,11]. A founder mutation, c.760C>T (p.R254X), in Chinese patients has a carrier rate of 1 in 125 [22]. To improve the newborn screening of citrin deficiency and CUD, we employed hotspot mutations analysis as the second-tier tests for these two diseases.

## Methods

### Newborn screening

Newborn screening was performed at the National Taiwan University Hospital Newborn Screening Center. Both screening and diagnostic cutoff values were set for citrulline and free carnitine to screen for citrin deficiency and CUD, respectively. Newborns with an initial screening value that exceeded the diagnostic cutoff were requested to participate in a confirmation test at our hospital. Newborns with an initial screening value not exceeding the diagnostic cutoff but equal to or exceeding the screening cutoff (inconclusive cases) were requested for a repeat DBS screening and enrollment in the second-tier molecular testing. Newborns with an abnormal repeat DBS result were also requested for a confirmation test at our hospital. Newborns with an abnormal result for galactosemia (screened by total galactose concentration), homocystinuria (screened by methionine concentration), or tyrosinemia (screened by tyrosine concentration) were also enrolled in the second-tier molecular testing for citrin deficiency. This study was approved by the Institute Review Board of the National Taiwan University Hospital. Written, informed consent for participation in the study was obtained from a parent of each participant.

### Molecular testing for citrin deficiency

DNA was extracted from one 3.2-mm punch from each DBS sample using Generation DNA Purification Solution and Generation DNA Elution Solution (Qiagen, Valencia, CA.). For citrin deficiency, we designed two allele-specific PCRs for the c.851_854del mutation (common left primer 5'-GTTAGGAGGAGGGCAGCAA, wild-type right primer 5'-CAATGTCTGCTAAGGTCATA, and mutant right primer 5'-CAATGTCTGCTAAGGTCGTC) and the IVS6+5 G > A mutation (common left primer 5'-TACAACTGGAGCACGCAAAG, wild-type right primer 5'-TCATTAGGGCAAGTTACAAC, and mutant right primer 5'-TCATTAGGGCAAGTTACAAT). The third PCR was designed to detect the c.1638_1660dup23 mutation (left primer 5'-TGTTGTGTCTCTRCCTCCTGCAGG, right primer 5'-GCAGTCTATCACTCCGCTGT). All three PCR products were subsequently analyzed using agarose gel electrophoresis and reconfirmed via direct sequencing with a sequencing primer set. Primer details will be provided upon request.

### Molecular testing for CUD

To detect the p.R254X mutation, we amplified exon 4 of the *SLC25A13* gene with primers 5'-CTCGCTGTTTTCTTGTCTG and 5'-TCTATGCTTCCTGTCTCTG. The PCR product was then digested with *Dde*I and analyzed using agarose gel electrophoresis. The resulting fragment for the wild-type sequence was 393 bp in length, whereas the fragments for the mutated sequence were 184 and 209 bp in length. Positive cases were reconfirmed by direct sequencing using a sequencing primer set.

## Results

### Screening for citrin deficiency

**Figure 1:**
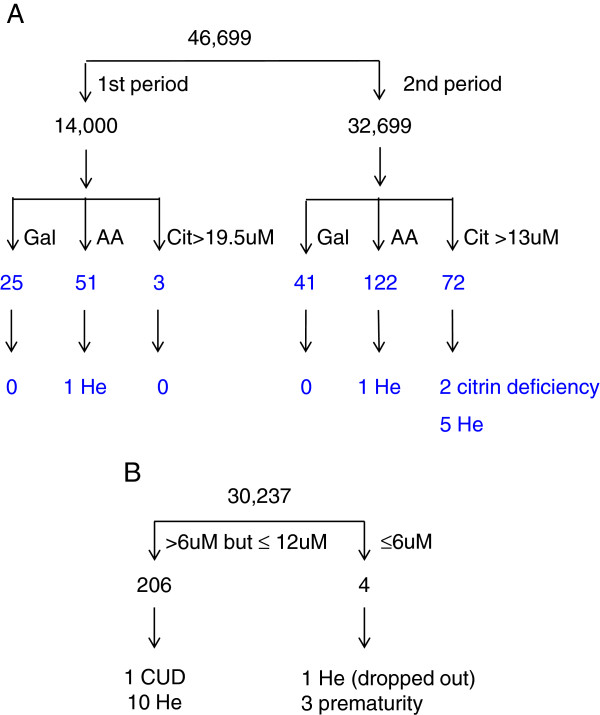
**Results of citrullinemia and carnitine uptake defects screening. **The study population included 46,699 newborns screened for citrullinemia (**A**) and 30,237 for carnitine uptake defects (**B**). (**A**) A total of 314 newborns were enrolled in the second-tier screening due to abnormal (but below the diagnostic cutoff) concentrations of total galactose, methionine, tyrosine, or citrulline in the initial dried blood spots. Gal: abnormal galactosemia screening results; AA: abnormal homocystinuria or tyrosinemia screening results; He: heterozygotes for *citrin* mutations. (**B**) A total of 210 newborns were enrolled in the second-tier screening due to low free carnitine concentration; among these, 4 exceeded the diagnostic cutoff. CUD: carnitine uptake defects; He: heterozygotes for p.R254X mutation.

During a 9-month period, the screening cutoff value was initially set at 19.5 μM, equal to 6 standard deviations (SD) above the population mean (mean=6.54, 1 SD=2.16) (first period), and this value was later reduced to 13 μM (equal to mean+3 SD) (second period). In 46 699 screened newborns, no cases exceeded the diagnostic cutoff of 110 μM, and 314 DBS samples (0.7%) were subjected to the second-tier testing due to abnormal levels of total galactose, methionine, tyrosine, or citrulline (Figure 1A).

Seven DBS samples with elevated citrulline levels and two with normal citrulline but abnormal methionine/tyrosine levels were identified as positive by the second-tier testing: two had the c.851_854del /IVS6+5 G > A compound heterozygous mutation, six had the c.851_854del heterozygous mutation, and one had the c.1638_1660dup23 heterozygous mutation (Table 1). Six of the newborns lived in the northern region of Taiwan, and three lived in the southern region; therefore, geographic clustering of this disease is unlikely. Of the DBS samples that demonstrated abnormal galactose concentrations during the initial screening, none were identified as positive for citrin deficiency during the second-tier testing. Two of the four subjects with the c.851_854del heterozygous mutation were found to have persistently elevated citrulline levels, but full sequencing of the *SLC25A13* gene showed no other mutations, and these subjects were classified as carriers of citrin deficiency. In total, two citrin deficient patients were identified; thus, the incidence of citrin deficiency in this cohort was approximately 1:23 350. The carrier rates for the first and second periods were 1:80 and 1:24, respectively. The predicted incidences were approximately 1:25 000 and 1:2 200, respectively.

**Table 1:** Newborns with a positive second-tier screening for citrin deficiency

**No.**	**First DBS citrulline (μM)**	**Mutation analysis**	**Diagnosis**
1	17.31	c.851_854del /IVS6+5 G>A	NICCD
2	15.5	c.851_854del /IVS6+5 G>A	NICCD
3	18.89	c.851_854del He	Negative
4	18.36	c.851_854del He	Negative
5	17.16	c.851_854del He	Negative
6	15.06	c.851_854del He	Negative
7	13.76	c.1638_1660dup23 He	Negative
8	5.03	c.851_854del He	Negative
9	5.01	c.851_854del He	Negative

NICCD: neonatal intrahepatic cholestasis caused by citrin deficiency.

### Screening for CUD

During a 6-month period, 30 237 newborns were screened for CUD (Figure 1B). Four newborns had free carnitine levels that were lower than the diagnostic cutoff of 6.0 μM (representing the bottom 0.01% of the population; population mean=24.46; 1 SD=7.52): three were premature babies that later proved to be normal, and one (with a heterozygous p.R254X mutation) refused the confirmatory test and was classified as a carrier (Table 2). A total of 206 newborns (0.7%) had inconclusive free carnitine levels (free carnitine≤12 μM, representing the bottom 1.8% of the population) and were enrolled for CUD second-tier molecular testing. The second-tier testing identified 10 heterozygotes with the p.R254X mutation and one subject with a compound heterozygous p.R254X/p.F17L mutation (Table 2). The 10 heterozygous newborns had normal free carnitine levels following second DBS testing. Therefore, in this cohort, the incidence of CUD was approximately 1:30 000 or greater, and the carrier rate for the p.R254X mutation in newborns with low initial DBS free carnitine levels was 1:19. The predicted incidence of CUD in this population is approximately 1:1 400.

**Table 2:** Newborns with a positive second-tier screening for CUD

**No.**	**First DBS free carnitine (μM)**	**p.R254X screening**	**Diagnosis**
Positive 1^st ^screening			
1	5.94	+	Lost to follow-up
2	5.23	-	Prematurity
3	3.27	-	Prematurity
4	4.51	-	Prematurity
Inconclusive 1^st ^screening			
1	7.90	+	Normal
2	10.20	+	Normal
3	11.70	+	Normal
4	11.36	+	Normal
5	11.91	+	Normal
6	7.65	+	Normal
7	10.26	+	Normal
8	7.36	+	Normal
9	8.50	+	Normal
10	10.32	+	Normal
11	6.84	+	CUD (p.R254X/p.F17L)

## Discussion

In this study, we sought to improve the performance of newborn screening for citrin deficiency and CUD. Newborn screening programs must determine cutoff values [2] to identify suspicious patients while minimizing false positives. However, patients with citrin deficiency may have nearly normal blood citrulline levels; similarly, patients with CUD may have normal carnitine levels due to placental transportation. Therefore, our strategy was to set a screening cutoff and a diagnostic cutoff, so we could test the inconclusive cases using a second-tier mutation analysis. We demonstrated that this strategy can improve the sensitivity of screening without increasing the false-positive rate.

In current practice, a successful molecular screening approach depends on the presence of founder or hotspot mutations. In citrin deficiency, the three mutations we screened (c.851_854del, c.1638_1660dup23, and IVS6+ 5 G>A) represent 95% of all mutations in Taiwanese patients [23] and 98% of all mutations in carrier screening [21]. In CUD, the p.R254X mutation accounts for 50% of all mutated alleles in clinical cases, but the prevalence of this mutation is lower in asymptomatic mothers and in screened newborns [17]. Therefore, to improve the performance of the screening program, we decided to subject these mutations to the second-tier testing.

Adding a second-tier mutation test can improve screening performance. In citrin deficiency, the two cases that we identified would have been missed by our original screening cutoff (19.5 μM), but we identified these cases after lowering the screening cutoff. The incidence of citrin deficiency derived from the current study (1:23 350) is close to the incidence (1:16 900) calculated from a mutation carrier rate of 1:70 [24]. From our study, in the group of newborns with high citrulline, methionine, tyrosine, or galactose, the carrier rates (i.e., 1:80 for the first period and 1:24 for the second period) were not significantly different from those identified in the previous study [24] or from those found in another study that included 479 healthy controls from Southern Taiwan [25]. The carriers that we detected in this study with elevated citrulline levels might harbor another *SLC25A13* mutation, or heterozygous carriers may even have slightly increased citrulline levels. Nevertheless, we would likely continue to miss some cases in which the subjects have normal citrulline levels at the newborn stage and only later present with cholestasis.

In CUD, the population carrier rate for p.R254X was shown to be 1:125, which suggests an incidence of 1:62 500 [22]. This mutation represented 50% of all mutations identified in clinically diagnosed patients and only 30% of mutations identified by newborn screening [17], which suggests that the actual incidence of CUD may be much higher. The CUD patient identified during this study was a compound heterozygote, which had an incidence of approximately 1 in 30 000 during this study period. Although this patient might have been identified without a second-tier mutation testing, not utilizing a second-tier mutation test approach would have required retesting 206 newborns (0.7%). Additionally, in the group of newborns exhibiting borderline low free carnitine, the carrier rate of the p.R254X mutation was 1:19 (11/206). Because 10 of the p.R254X heterozygous newborns were found to have normal free carnitine levels in the second screen, we speculate that those were true heterozygous carriers, not patients. This is a reasonable assumption because carriers of CUD may present with slightly low free carnitine, and our selection biased the carrier rate of this mutation. Although carnitine administration ameliorates all symptoms of CUD, we continue to screen patients using a second screen, but we do not apply this new algorithm using a second-tier test due to cost.

The major limitation of this study was our selection of patients based on an abnormal initial screening. If the levels of citrulline, galactose, methionine, tyrosine or carnitine were normal, the second-tier testing would not have been performed. A rapid genetic test for citrin deficiency performed by screening for 11 common mutations of *SLC25A13* has been developed [26], but further validation using a larger scale of dried blood samples is necessary prior to the clinical application of this technique. In addition, the low incidence of these diseases makes it difficult to attain a case number of sufficient size for a statistically significant conclusion. Lastly, the cost of molecular testing may hinder the availability of genetic screening programs. However, if molecular screening for other diseases has previously been conducted, a second-tier molecular test may be easier to perform because the cost would be minimized by previous DNA extraction.

In Taiwan, we initiated newborn screening for citrin deficiency and CUD in 2001. Newborn screening for citrin deficiency has been shown to result in early treatment for newborns suffering from NICCD, or preventing erroneous management if CTLN2 develops in these patients. The plasma citrulline level is the current marker for newborn screening of NICCD, but this marker is not sensitive enough to detect all patients with NICCD: we have encountered patients with normal newborn screening results for whom a diagnosis could not be made before the occurrence of liver failure. Similarly, we have shown that newborn screening leads to early treatment for newborns suffering from CUD and that carnitine administration prevents the occurrence of symptoms in these patients. Plasma carnitine is the current marker for CUD, but both false-positive and false-negative results occur: we diagnosed one CUD patient who had recurrent hyperammonemia but whose newborn screening, performed by another screening center, was normal. Therefore, while it is very important to maintain current screening for citrin deficiency and CUD, there is an urgent need to find a method to further improve these screening tests.

## Conclusion

We demonstrate the improvement of newborn screening for citrin deficiency and carnitine uptake defects using a second-tier mutation analysis. Further adjustments to improve the sensitivity of this test are warranted.

### Key notes

The addition of a second-tier mutation testing can improve newborn screening sensitivity for citrin deficiency and carnitine uptake defect.

## Competing interests

The authors declare that they have no competing interests.

## Authors’ contributions

LYW and NIC performed the analyses in this study. PWC conducted the sample collection and preparation. SCC designed and validated the analytical methods. WLH and YHC designed the study and drafted the manuscript. NCL verified genotyping data and collected samples from patients. All authors read and approved the final manuscript.

## Pre-publication history

The pre-publication history for this paper can be accessed here:

http://www.biomedcentral.com/1471-2350/14/24/prepub
